# Siwalik sabrecats: review and revised diagnosis of *Megantereon* fossils from the foothills of the Himalaya

**DOI:** 10.1098/rsos.231788

**Published:** 2024-05-08

**Authors:** Christopher M. Stimpson

**Affiliations:** ^1^ Oxford University Museum of Natural History, Oxford, OX1 3PW , UK

**Keywords:** Siwaliks, *Megantereon*, sabretooth cat, morphometrics

## Abstract

The diagnosis of different fossil taxa in small collections from disparate geographical and temporal contexts is a common challenge in palaeontology. The likely number of morphospecies of the extinct sabretooth cat *Megantereon* is a classic example and subject of long-standing debate. While analyses of global fossil collections have provided insights and hypotheses, specimens from the foothills of the Himalaya—the Siwaliks—have been overlooked in recent treatments due to poor characterization and a confused taxonomic history. Here, this oversight is addressed. Craniodental fossils from the Siwaliks are revisited and their taxonomic status is reviewed. Morphological and metric characteristics are described, and qualitative and quantitative comparisons with congenerics are performed with published descriptions and datasets. The Lower Pleistocene Siwalik *Megantereon* are among the largest known forms in the genus. Advanced characteristics include reduced upper third premolars and long but comparatively narrow carnassial teeth. While dietary specialism can constrain morphological diversity, statistical analyses, including controls for body size effects, detected significant metric differences in the mandibles in comparison with congenerics. Within current paradigms, the status of *Megantereon falconeri* as a distinct morphospecies is upheld. A revised diagnosis is provided and the taxonomic affinities of *M. falconeri* are considered.

## 1. Introduction

Taxonomy is a fundamental concept and a foundation of practice in the biological sciences [1]. In palaeontology, the classical focus of taxonomic work is morphology and adheres to a morphological species concept [2]. The establishment of robust criteria to diagnose different morphospecies in relatively small numbers of specimens from disparate geographical and temporal contexts is, however, an intrinsic challenge [3]. The sabretooth cat genus *Megantereon* (Croizet and Jobert, 1828) is a classic example; the taxonomic history of these extinct hypercarnivores has been a troubled one [4] and the number of species within the genus remains a contentious subject [5]. Fossils of carnivorous mammals can present particular challenges for taxonomic differentiation and diagnosis. Firstly, due to energetic constraints, carnivores are relatively rare in the landscape [6] and this scarcity, outside of exceptional taphonomic settings [7], tends to manifest in the fossil record as relatively small sample sizes [8]. Secondly, these jaguar-sized sabrecats were highly specialized predators and their most commonly described fossils are craniodental remains [9]. While specialization does not necessarily affect taxonomic diversity, it can impose constraints on morphological diversity in elements of the dentition (e.g. in cheek teeth) [10].

**Figure 1.**
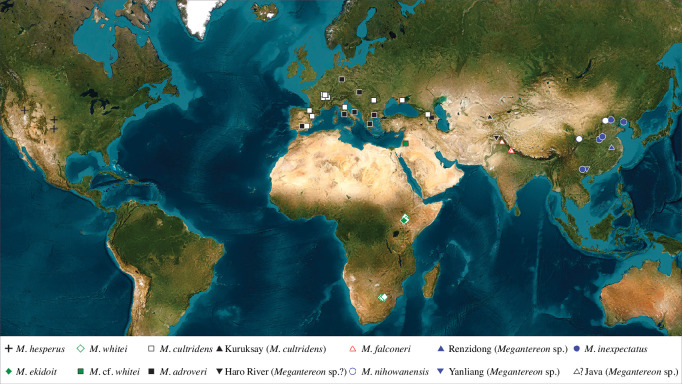
Geographical distribution of fossils of *Megantereon* spp. Late Pliocene/Lower Pleistocene: *M. cultridens*, Kuruksay *Megantereon*, *M. whitei*, *M. ekidoit*, *M. hesperus*; Lower Pleistocene: *M. nihowanensis*, Renzidong and Yanliang *Megantereon*, Java *Megantereon*, Siwalik *Megantereon*; later Lower Pleistocene: *M. adroveri*; Middle Pleistocene: *M. inexpectatus*. Taxonomic conventions adapted from [5].

Multiple studies have revisited taxonomic relationships within *Megantereon* and yielded insights and competing hypotheses [5,9,11–23]. There has, however, been an omission in more recent reviews [17]. Despite being among the longest-known fossils of *Megantereon*, specimens collected in the foothills of the Himalaya—the so-called Siwaliks—have not been considered in any detail in recent treatments. The last review of the genus to incorporate data from the Siwalik *Megantereon* fossils was published in the late twentieth century [12] and the last dedicated re-examination took place over 40 years ago [22]. These specimens represent the sole source of data for South Asia (figure 1) and their absence has been conspicuous [15], particularly in light of the recovery of well-preserved *Megantereon* fossils in China [16,17,20].

Here, Siwalik *Megantereon* fossils are revisited with the following aims: (i) to review their morphological characteristics; (ii) to produce and make available a comprehensive metric dataset; and (iii) to perform comparisons with congenerics using published data and descriptions to reassess their taxonomic status and affinities.

### 1.1. Temporal, taxonomic and geographical contexts of *Megantereon*

Fossils of *Megantereon* range in age from the Pliocene, from approximately 3.6 million years ago (Ma), into the Lower Pleistocene, 2.58–0.77 Ma, until the Middle Pleistocene approximately 0.7–0.6 Ma (figure 1). There have been at least 12 named species but not all are accepted. This issue remains contentious and useful summaries can be found in [5,15]; the taxonomic framework that was employed in the present study was adapted from these works. This adaptation reflects practical concessions to preserve geographical and temporal variation, as much as a recognition of potential taxonomic diversity, for the purposes of comparative analysis.

There have been proposals that there is just one species in the genus, *M. cultridens* (Cuvier, 1824), and that metric and morphological characterisitcs vary due to sexual dimorphism and geographic and temporal trends [11]. It is now accepted, however, that there are at least two species within the genus [9]. Here, *M. cultridens* is regarded as a western Eurasian species of the late Pliocene and Lower Pleistocene. This species may have comprised two forms: an early, relatively small form and a later, larger and more derived form [5,13]. The second accepted species, *M. whitei* (Broom 1937), is described from Africa and is differentiated from *M. cultridens* by a reduction in absolute and relative dimensions of dental elements [5,9]. Another African species, *M. ekidoit* (Werdelin and Lewis, 2000), however, has also been described [23] and represents one of the earliest records of the genus in the Old World.

In North America, rare fossils with primitive characters have been named *M. hesperus* (Gazin, 1933) [24], although early specimens have been reclassified and placed in a separate genus, *Rhizosmilodon* [25].

During the Lower Pleistocene, a species with derived characteristics and distinct from *M. cultridens* appeared in Eurasia. Opinions have varied as to its identity. It has been proposed that this form reflected a northward dispersal of *M. whitei* from Africa [12], although known fossils of this taxon are geographically limited to the south and east of the continent. *Megantereon* cf. *whitei* is also tentatively reported from 'Ubeidiya in Israel, although diagnostic material is scant [26]. An alternative scenario is that the advanced form evolved *in situ* from *M. cultridens* [15], designated as *M. adroveri* (Pons Moya, 1987) [19]. The latter convention is retained here.

Specimens from Kuruksay in Tajikistan are rare Central Asian records. These fossils were initially classified as a novel species, *M. vakhscensis* (Sharapov, 1986), but this has not been generally accepted [13]. The Kuruksay specimens have variously been regarded as conspecific with *M. cultridens* [9] or with the Siwalik specimens [13]; they appear indistinguishable from smaller forms of the former taxon [9]. In Southeast Asia, canines attributed to *Megantereon* are described from the Pleistocene of Java [27] although their provenance is uncertain [28].

Remarkably well-preserved fossils of *Megantereon* have been recovered in China and there are several reported species. While some authorities regard some or all as likely synonymous with the Siwalik specimens [5,13,29], the status and affinities of the Lower Pleistocene taxa from East Asia are complex issues. The name *Megantereon megantereon* (a synonym of *M. cultridens* [18]) was resurrected following [30], to account for Lower Pleistocene specimens from Renzidong Caves [31] (here, referred to as the Renzidong *Megantereon* sp.) and to distinguish them from *M. nihowanensis* (Teilhard de Chardin and Piveteau, 1930) a species that reportedly has close affinities with *M. cultridens* ([9,32]; but see [33]). *Megantereon megantereon* has also incorporated a small, Lower Pleistocene specimen from Yanliang Cave, Guangxi [17] (here, referred to as the Yanliang *Megantereon* sp.). This specimen had also been attributed to a novel species, ‘*Megantereon microta*’ [32], although this diagnosis requires clarification [16]. A later, relatively large species, *M. inexpectatus* (Teilhard de Chardin, 1939), is recorded from Middle Pleistocene contexts and represents the last known occurrence of the genus in the Old World [17].

### 1.2. A brief history of Siwalik *Megantereon*

The fossil-bearing Siwalik group and associated Neogene-Quaternary deposits of the Himalayan foreland basin extend between the Indus and Irrawaddy rivers, in the foothill and valley systems to the southwest of the Himalaya. These formations comprise strata that date from the Miocene (approx. 23 Ma) to the Lower Pleistocene (2.58–0.78 Ma). Of the seven *Megantereon* fossils that are considered in this study, six were collected during an intensive period of collection in the early nineteenth century in India, which began in November 1834 [34]. They have since been associated with Upper Siwalik deposits attributed to the Pinjor faunal zone and thus date to the Lower Pleistocene [35], 2.58–0.78 Ma. Measurements of a mandible that was recovered in association with Upper Siwalik deposits in the Pabbi Hills in Pakistan, also from the Pinjor faunal zone, are also incorporated [36]. Fragmentary specimens of an upper canine and a mandible from Haro River quarry in northern Pakistan have also been tentatively assigned to the genus and may represent a very large form [37] but require confirmation.

Fossils of carnivorous mammals from the Siwaliks are diverse but rare and the status of many taxa is unclear [38,39]. The omission of Siwalik fossils from recent discussions of *Megantereon* is reasonably explained by the fact that treatments of machairodont cats in this collection have ‘been confused and have caused confusion’ [11, p. 126] and they have remained poorly characterized [5]. In the formative works on Siwalik machairodonts, specific diagnoses also incorporated fossils of *Homotherium,* and, as is frequently the case in Siwalik fossil taxa, nomenclature has varied between authorities with a lack of clarity regarding type specimens [40]. Comments in Pilgrim [41] are a clear review of early works and the basis of the following brief review.

In 1846, Siwalik *Megantereon* and *Homotherium* specimens, now in the Natural History Museum UK (NHMUK), were referenced by Owen [42] as ‘*Machaerodus*’ without diagnosis or type. A cursory diagnosis of ‘*Megantereon falconeri*’ by Pomel [43] followed in 1853, based on the examination of *Homotherium* fossils, which included a maxilla fragment of a juvenile (NHMUK PV OR 16350) and likely an adult mandible (NHMUK PV OR 48436) in addition to two mandibles of *Megantereon* (NHMUK PV OR 16557 and NHMUK PV OR 16554). No type was specified. In 1862, Gaudry [44] retained Pomel’s specific designation but reverted Owen’s generic classification to reference ‘*Machaerodus falconeri*’. In Falconer’s *Palaeontological Memoirs* [34], specimens from both genera (*Homotherium* NHMUK PV OR 16350 and *Megantereon* NHMUK PV OR 16557) are figured in Plate XXV as ‘*Drepanodon sivalensis*’. No type was cited. Bose [45] discussed the specimens examined by Owen and referred to ‘*Machaerodus sivalensis’* but made no reference to a type. While ‘*Machaerodus sivalensis’* incorporated a specimen that would ultimately be revealed as *Homotherium* [22], Bose did recognize the presence of a second distinct taxon and named it ‘*Machaerodus palaeindicus’*.

In 1884, Lydekker’s [46] descriptions of ‘*Machaerodus sivalensis*’ included: (i) a near complete mandible (NMI NH F18579) that was purchased from local collectors by WE Baker and HM Durand and now in the National Museum of Ireland (NMI); (ii) a mandible in the collections of the Geological Survey of India (GSI D-100) that was collected in the vicinity of Roorkee (‘Rúrki district’); (iii) a fragment of a left maxilla in the NHMUK collection (NHMUK PV OR 39730); and (iv) a mandible (NHMUK PV OR 16557), also in the NHMUK collection, which was cited as type. Lydekker’s description of ‘*Machaerodus sivalensis*’ retained the juvenile *Homotherium* maxilla (NHMUK PV OR 16350) but, following Bose, the presence of a larger taxon in the Siwalik assemblages was recognized as *M. palaeindicus*. Matthew [47] echoed Lydekker’s work but used ‘*Meganthereon falconeri’* rather than *M. sivalensis*. Specimen NHMUK PV OR 16557 was affirmed as lectotype and a further mandible (NHMUK PV OR 48929) was added to the inventory for the taxon. Pilgrim [41] provided a detailed review of specimens but the diagnosis of ‘*Meganthereon* (?) *falconeri*’ was still muddied by the retention of the juvenile *Homotherium* maxilla. Colbert [48] referenced *Megantereon falconeri* but restricted discussion to the upper dentition and referred readers to [47].

A brief study of the Siwalik material by Petter and Howell [22] was a critical step towards clarity. By examination of the maxillae and length measurements of the fourth lower premolar and first lower molar, the fossils of *Homotherium* were separated from *Megantereon* and the presence of both genera in the Pinjor faunal zone of Upper Siwaliks was confirmed. While these authors indicated that the Siwalik specimens showed some distinct characteristics, they cautioned that it was not possible to separate them from *M. cultridens*. Their recommendation, however, was that the Siwalik specimens should retain a specific distinction [22]. While the description and taxonomic treatment of the Siwalik *Megantereon* has been confused, and diagnoses were based on specimens from different genera, the name *M. falconeri* (Pomel 1853) has priority [49].

## 2. Material and methods

**Figure 2.**
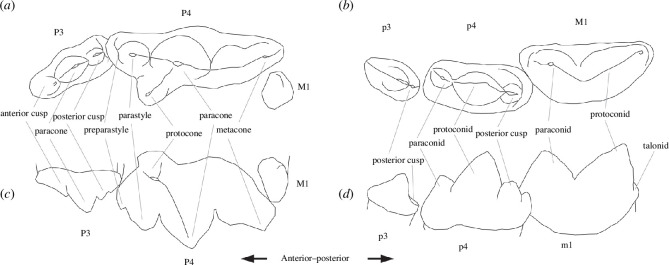
Generalized sabrecat dentition to show characters in cheek teeth: (*a*) left upper, occlusal view; (*b*) left lower, occlusal view; (*c*) right upper, lingual aspect; and (*d*) left lower, buccal aspect. Adapted from [50].

**Figure 3.**
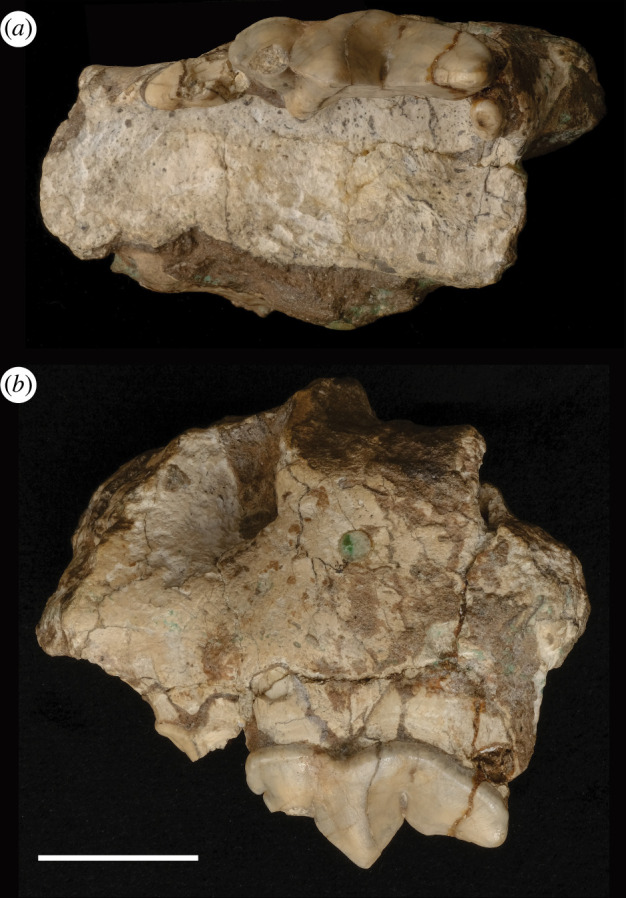
NHMUK PV OR 39730 left maxilla of *M. falconeri*; (*a*) occlusal view and (*b*) buccal aspect. Scale bar = 20 mm.

**Figure 4.**
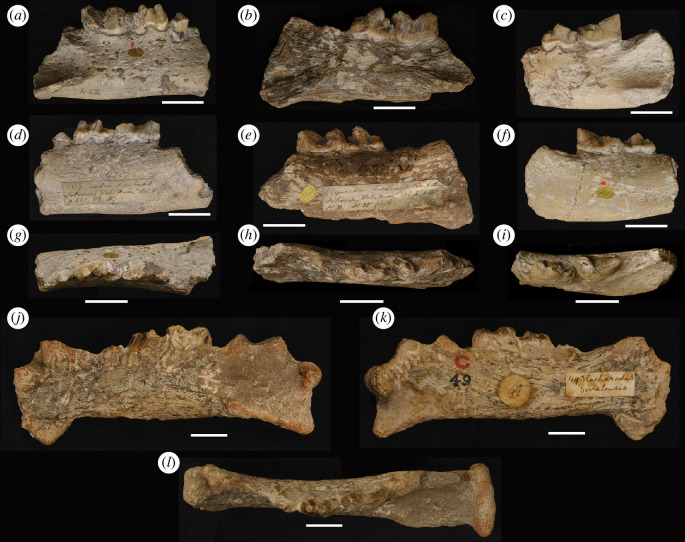
Buccal, lingual and occlusal aspects of mandibles of *M. falconeri*: NHMUK PV OR 16554 (*a*, *d, g*); NHMUK PV OR 16557 (*b*, *e*, *h*); NHMUK PV OR 48929 (*c, f, i*); NMI NH F18579 (*j–l*). Scale bars = 20 mm.

Descriptions of dental characters and location terminology are adapted from [29] and [50] and are annotated in figure 2. Seven *Megantereon* fossils from the Siwaliks were considered. One maxilla (figure 3) and four mandibles (figure 4) were examined directly. Published measurements from two mandibles were also incorporated: 674 GB 21 recovered during palaeontological investigations of the Pabbi Hills in Northern Pakistan in the late twentieth century [36] and a nineteenth-century specimen, GSI D-100 from the Geological Survey of India [41] (table 1). The NHMUK specimens derived from the collection of Hugh Proby Cautley and were presented to the British Museum in 1842 [51]. The NMI mandible was purchased together with an array of Siwalik fossils [52] from a Dr Beatty by the Museum of the Royal Dublin Society (N. Monaghan 2023, personal communication). This material included fossils from the so-called ‘Dadúpúr collection’ of Baker and Durand [53].

**Table 1.** Specimen details and measurements (mm) of *M. falconeri* fossils compared with previous studies.

maxilla	P3L	P3W	P4L	P4W	M1L	M1W	source
NHMUK PV OR 39730	11.38	6.15	33.17	11.6	4.34	6.22	this study
Lydekker, 1884	*11.43*	*—*	*33.02*	*—*	*3.81*	*6.86*	[46]
Pilgrim, 1933	***12.5***	*6.5*	*33*	*12.3*	*4.5*	***8***	[41]

**mandibles**	**p3L**	**p3W**	**p4L**	**p4W**	**m1L**	**m1W**	**JDm1**	**source**
NMI NH F18579	(~11.5)	(~5.5)	21.84	10.54	25.7	11.56	36.57	this study
Lydekker, 1884	*11.18*	*—*	***23.88***	*10.16*	*25.91*	*—*	*35.81*	[46]
NHMUK PV OR 16554	9.1	5.52	20.19	9.74	22.4	10.05	34.8	this study
Lydekker, 1884	*9.4*	*—*	*20.32*	*—*	*22.86*	*—*	*34.54*	[46]
Turner, 2004	*9*	*5.5*	*21*	*9.5*	*22.7*	*9.8*	*—*	[36]
NHMUK PV OR 16557	**—**	—	20.97	9.6	24.57	10.36	>32	this study
Lydekker, 1884	*—*	*—*	*21.59*	*—*	*24.9*		29.97*	[46]
Pilgrim, 1933	*—*	*—*	*—*	*—*	*24.5*	*11*	*—*	[41]
Turner, 2004	*—*	*—*	*20.7*	*9.7*	*24.5*	*10.3*	*—*	[36]
NHMUK PV OR 48929	**—**	—	18.84	9	22.42	9.84	33.98	this study
GSI D-100	8	6.3	21	10.5	26	13	33	[41]
674 GB 21	8.1	5	21.5	8.8	25.5	12.2	—	[36]

*Notes:* Differences greater than 1 mm are shown in bold. Values in italics are from previous studies. P3, third upper premolar; P4, fourth upper premolar; M1, first upper molar; p3, third lower premolar; p4, fourth lower premolar; m1, first lower molar; JDm1, depth of mandibular corpus anterior of the m1.

L, length; W, width.

Metric conventions follow [9] and are defined where they are employed. All measurements were performed 10 times with analogue dial callipers to the nearest 0.01 mm. Mean values were used in analyses and were compared, where available, with previous metric treatments of the Siwalik specimens [36,41,46]; (table 1).

Morphological descriptions and metric data of *Megantereon* fossils were compiled from published sources [9,11–21,23,30–32]; named species of *Megantereon* were established *a priori* (electronic supplementary material, table S1). Taxonomic affinities of the Renzidong and Yanliang fossils were considered during analyses. To the author’s knowledge, all measurements were derived from specimens with adult dentition; specimens with deciduous dentition are exceptionally rare [54]. Measurements were audited for redundancy, inclusion of data from broken or deformed specimens, from casts and measurements of alveoli. All comparative data are included in electronic supplementary material, table S1.

Together with sexual size dimorphism, geographical and temporal homogenization are potential confounding factors in the metric analyses of fossil felids [55]. This study proceeded with the assumption that the samples of metric characters derived from non-normal distributions and statistical examinations employed non-parametric procedures. Multi- and bi-variate datasets were compared using a one-way PERMANOVA [56] performed in PAST 4.13 [57] with a stated *p* = 0.05; corrected *p*-values were derived using Holm’s sequential Bonferroni procedure to control for inflation in type I error rates in multiple comparisons [58]. Statistical testing examined raw measurements and variables adjusted for allometric effects (below). Sample size in comparative data dictated the taxonomic resolution of comparisons. Minimally, the Siwalik specimens were compared at the genus level and/or with datasets parsed into two groups in recognition of the two accepted morphotypes. Group 1 comprised specimens of *M. cultridens*, *M. nihowanensis*, *M. inexpectatus* and specimens from Kuruksay, Renzidong and Yanliang; data from the Siwalik specimens were incorporated into this group for the purposes of investigating potential body size effects (below). Group 2 included African and derived European morphotypes, *M. whitei* and *M. adroveri*, respectively. Standalone comparisons with datasets for *M. cultridens* were also performed where sample size permitted.

Diagnostic characters in craniodental fossils of *Megantereon* include size, shape and relative proportions. While size is adaptive, morphological differences can reflect allometric effects, particularly in felids [59]. Possible analytical controls for size effects have been the focus of considerable study and comment, as has criticism of various techniques put forward to compensate for them [60]. Ratios are commonly used in analyses of *Megantereon* fossils and seem intuitive but can be beset by analytical and interpretative problems due to allometric effects [61].

While the relationship between carnassial length and size is not as defined or as optimal as measures such as condylobasal length of the cranium [62], the lengths of these teeth are positively correlated with size and mass in felids [50,62,63]. Here, lengths of the carnassial teeth, the upper fourth premolar (P4) and the lower first molar (m1) were employed as proxies for body length. Potential size effects in log-transformed variables were explored via regression and the Spearman rank permutation test (*r*_s_) functions in PAST, where body size effects were implicated, a correction was applied based on Thorpe’s allometric formula [64] following the procedure described by [61],


(2.1) $$x_{adj}=log\left(x\right)-b\left[log\left(BL\right)-log\left(BL_{mean}\right)\right],$$

where *x*_adj_ is the variable of interest, BL is a standard measurement of size (here, length of the corresponding carnassial tooth), *b* is the slope of the relationship between log(*x*) and log(BL) and BL_mean_ is the mean of BL values.

The results of tests for body size effects are summarized in table 2; regressions and computations of size-corrected variables are included in electronic supplementary material, table S2. Summaries of non-parametric comparisons between sets of measurements and Holm’s sequential Bonferroni corrected *p* values are shown in table 3; test datasets, the products of statistical comparisons and calculations of Holm’s sequential Bonferroni corrected *p*-values are included in electronic supplementary material, table S3.

**Table 2.** Summary of results of ordinary least squares regression and non-parametric correlation (Spearman’s rank *r*_s_) for craniodental variables in *Megantereon*.

test	dataset	BL (proxy)	variable	*n*	*r* ^2^	*r*_s_	*p*
size effects—maxilla	genus	P4L	P3L	26	0.062	0.173	0.39
	group 1	P4L	P3L	20	0.027	−0.278	0.23
	genus	P4L	P3W	25	0.018	0.1	0.63
	group 1	P4L	P3W	20	0.084	−0.29	0.21
	genus	P4L	P4W	33	0.187	0.39	*0.03*
	group 1	P4L	P4W	23	0.0003	−0.13	0.55
	group 2	P4L	P4W	10	0.006	−0.231	0.52
size effects—mandible	genus	m1L	p3L	22	0.062	0.093	0.68
	group 1	m1L	p3L	19	0.319	−0.59	*0.02*
	genus	m1L	p4L	29	0.683	0.86	*<0.0001*
	group 1	m1L	p4L	21	0.684	0.85	*<0.0001*
	genus	m1L	p4W	29	0.74	0.85	*<0.0001*
	group 1	m1L	p4W	21	0.668	0.805	*<0.0001*
	genus	m1L	m1W	44	0.807	0.845	*<0.0001*
	group 1	m1L	m1W	29	0.833	0.925	*<0.0001*
	group 2	m1L	m1W	15	0.058	0.228	0.42
	genus	m1L	JDm1	22	0.146	0.428	*0.049*
	group 1	m1L	JDm1	15	0.199	0.479	0.07
	group 2	m1L	JDm1	7	0.082	0.09	0.85

*Notes:* Significant results are shown in italics.

**Table 3.** Summary of results of non-parametric comparisons (one-way PERMANOVA; *p* = 0.05) of raw and size-adjusted dental metrics of *M. falconeri* and congenerics with Holm’s sequential Bonferroni corrected *p*-values.

test variables	datasets (*n*)	*F*	*p*	corr. *p*
raw p4L, p4W	*M. falconeri* (*n* = 6), group 1 (*n* = 15)	8.372	0.008	*0.048*
raw p4L, p4W	*M. falcon*e*ri* (*n* = 6), group 2 (*n* = 8)	23.34	0.0004	*0.006*
raw p4L, p4W	*M. falconeri* (*n* = 6), *M. cultridens* (*n* = 11)	9.358	0.0063	*0.0441*
p4L_adj_, p4W_adj_	*M. falconeri* (*n* = 6), group 1 (*n* = 15)	0.6792	0.4933	0.9866
p4L_adj_, p4W_adj_	*M. falconeri* (*n* = 6), *M. cultridens* (*n* = 11)	0.4783	0.6037	0.6037
raw m1L, m1W	*M. falconeri* (*n* = 6), group 1 (*n* = 23)	9.148	0.0047	*0.0376*
raw m1L, m1W	*M. falconeri* (*n* = 6), group 2 (*n* = 15)	80.86	0.0002	*0.0032*
raw m1L, m1W	*M. falcone*ri (*n* = 6), *M. cultridens* (*n* = 17)	10.79	0.0035	*0.035*
logm1L, m1W_adj_	*M. falconeri* (*n* = 6), group 1 (*n* = 23)	10.85	0.0014	*0.0182*
logm1L, m1W_adj_	*M. falconeri* (*n* = 6), *M. cultridens* (*n* = 17)	12.88	0.0007	*0.0098*
raw m1L, m1W, JDm1	*M. falconeri* (*n* = 4), *Megantereon* (*n* = 18)	7.838	0.0041	*0.0369*
raw m1L, m1W, JDm1	*M. falconeri* (*n* = 4), group 1 (*n* = 11)	5.193	0.025	0.10
raw m1L, m1W, JDm1	*M. falconeri* (*n* = 4), group 2 (*n* = 7)	21.84	0.003	*0.033*
logm1L, m1W_adj_, JDm1_adj_	*M. falconeri* (*n* = 4), *Megantereon* (*n* = 18)	3.826	0.016	0.08
logm1L, m1W_adj_, JDm1_adj_	*M. falconeri* (*n* = 4), group 1 (*n* = 11)	3.207	0.042	0.126
logm1L, m1W_adj_, JDm1_adj_	*M. falconeri* (*n* = 4), group 2 (*n* = 7)	19.16	0.0023	*0.0276*

*Notes:* Significant differences are shown in italics.

## 3. Results

### 3.1. Metric comparison with previous studies

The measurements in this study were largely consistent with previous works and within less than 1 mm; three measurements were less than or equal to 2 mm. Tooth breakage and the presence of matrix on mandible NMI NH F18579 obscures the interface between the lower fourth premolar (p4) and the first lower molar (m1); to be clear, the original measurements were in inches, but it appears that p4 length was over-measured by Lydekker [46]. In the maxilla, NHMUK PV OR 39730**,** Pilgrim [41] reports comparatively large values for the length of the upper third premolar (P3L) and width of the upper first molar (M1W). It is not possible to reconcile Petter and Howell’s [22] measurements with individual specimens, although reported ranges of the first lower molar length (22–26 mm) and fourth lower premolar length (18–23 mm) are consistent with previous reports and the present study (table 1).

### 3.2. Maxilla: description

NHMUK PV OR 39730 (figure 3) is a fragment of the left maxilla with upper third premolar (P3), upper fourth premolar (P4) and upper first molar (M1) *in situ*. The specimen is pale brown with dark mottling. The posterior edge of the canine alveolus is partially preserved. The infraorbital foramen appears relatively large. The P3 appears simple without developed accessory cusps; the paracone and the buccal side of tooth are broken. The P3 is reduced in proportion to the P4; the anterior cusp is poorly defined. The occlusal tip of the P4 parastyle is chipped but the remainder of this tooth is intact. There is a small but well-defined preparastyle on the anterior edge of the parastyle. The protocone of the P4 is distinct and is situated immediately posterior to the junction of the parastyle and the paracone. The protocone extends lingually but appears reduced. The M1 is also much reduced, is oval in shape and is shortened in the anteroposterior direction. There is a small but well-defined cusp. The angle between the lingual edges of the P3 and P4 is obtuse.

### 3.3. Maxilla: comparisons

**Figure 5.**
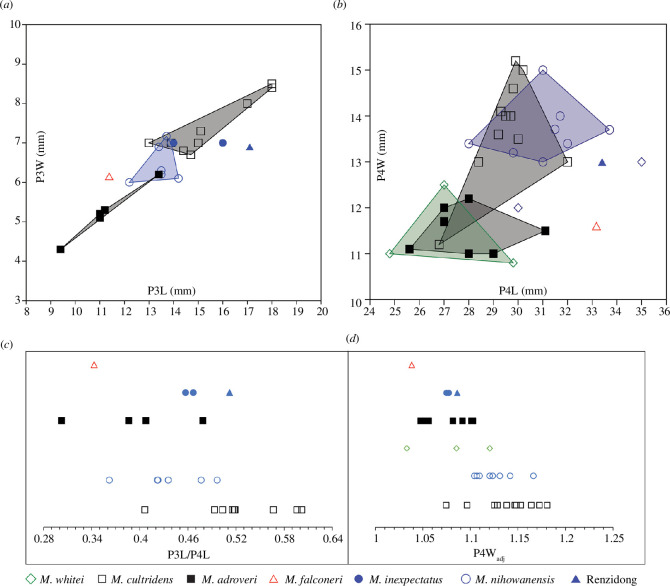
Metric comparisons of the upper dentition in *Megantereon* fossils: (*a*) bivariate plot of raw measurements of third upper premolar length (P3L) and width (P3W); (*b*) bivariate plot of raw measurements of fourth upper premolar length (P4L) and width (P4W); (*c*) the proportion of P3L to P4L as a ratio; and (*d*) size-adjusted ranges of P4W (P4Wadj).

The P3 is simple and is smaller in absolute dimensions than *M. cultridens*, *M. inexpectatus* and the Renzidong *Megantereon* and more similar to *M. adroveri* and *M. nihowanensis* (figure 5*a*). Investigation of body size effects did not indicate any clear relationship between size and the dimensions of the P3 (table 2). There was no indication of effects on P3 length (P3L) at the level of genus or in group 1; small sample size precluded the parsing and testing of the group 2 data. There was no indication of size effects on P3 width (P3W) across the genus, or in group 1.

While the relative proportions of the P3 to the P4 (expressed as a simple ratio P3L/P4L in figure 5*c* ) do show overlap between and variation within named species, the relative proportion of NHMUK PV OR 39730 indicates a marked reduction. It approaches the smaller end of the range of the data for *M. nihowanensis* and overlaps with equivalent values for *M. adroveri*. Conversely, the reduction of the P3 is less evident in *M. cultridens*, the small sample of *M. inexpectatus* and the Renzidong *Megantereon* (figure 5*c* ).

The presence of a preparastyle on the P4 appears to be a variable character in *Megantereon*. It is recorded in early specimens of *M. cultridens* from Perrier-les Etouaires [13] and St Vallier [30] and in East Asia, in a Lower Pleistocene specimen of *M. nihowanensis* (V 26878) and the Renzidong *Megantereon* (V 13042. 11) [14,31]. In absolute dimensions, the P4 of NHMUK PV OR 39730 is comparatively long but narrow (figure 5*b*) due to the reduced protocone. Regression of P4 length against width implied size effects in the genus; no relationship, however, was detected when these data were parsed as group 1 or group 2 (table 2). The size-adjusted value of the width of the upper fourth premolar (P4W_adj_) of NHMUK PV OR 39730 falls below the lower limits of those of the group 1 taxa and closest to *M. adroveri* and the small sample of *M. whitei*. In contrast, while there is variation, the protocone of the P4 in *M. cultridens* and *M. nihowanesis* is well developed [5,17] and reflected by larger values of P4W_adj_ (figure 5*d*).

Petter and Howell [22] regarded the P4 morphology of NHMUK PV OR 39730 to recall an example of *M. cultridens* from Olivola (IGF-4712); this skull is deformed, however, and only an approximate value for P4W was given in the original metric examination (‘*ca* 13’ [18]). The protocone of a small specimen of *M. cultridens* from Pardines (LP 18) also appears reduced. This specimen differs from conspecifics in absolute dimensions and appears as an outlier and is similar to *M. adroveri* (figure 5*b*). Amidst the group 1 morphotypes, the size-adjusted value of the Siwalik specimen (P4W_adj_) more closely matches *M. inexpectatus* (figure 5*d*) and the Renzidong *Megantereon*. The metric characteristics of the upper dentition in these taxa appear similar, although the sample size is small. While the protocone of the Renzidong specimen is more developed, a reduced protocone is regarded as a diagnostic character of *M. inexpectatus* [17].

### 3.4. Mandibles: descriptions

NHMUK PV OR 16554 is a right mandible (figure 4*a,d,g* ). A portion of the mandibular corpus is preserved and the third lower premolar (p3), fourth lower premolar (p4) and first lower molar (m1) are *in situ*. The specimen is pale and lacks major staining; mottled dark patches are present on the buccal side of the corpus. The anterior portion of the masseteric fossa is present, which is triangular and terminates at the midline of the m1. A foramen is present on the lingual side of the posterior end of the corpus. The p3 is simple, complete and appears to have a single root; a weakly developed posterior cusp is present. There is a very small p3–p4 diastema. The p4 is inclined to the posterior and overlaps with the buccal side of the anterior end of the m1. The paraconid is intact but the protoconid and a portion of the buccal side of the tooth are broken. The posterior cusp appears to have been well developed. The m1 has breaks to the paraconid and protoconid. No talonid is present.

NHMUK PV OR 16557 is a left mandible (figure 4*b,e,h* ). The specimen has a mottled brown appearance, exacerbated by tool marks and with black patches and stria, most noticeably on the lingual surface of the specimen. A portion of the mandibular corpus and mandibular flange, with foramen, is preserved. The mandibular flange appears to have been well developed and forms an angle of approximately 145° with the ventral edge of the corpus. The anterior end of the masseteric fossa terminates in line with the midline of the m1. The ventral edge of the corpus is only intact to the anterior side of the p4. The p3 is broken at the root. The p4 and m1 are *in situ*; the p4 overlaps with the buccal side of the m1. The p4 is high crowned. The buccal surface of the paraconid is chipped. The well-developed protoconid is complete, inclined to the posterior, with a well-developed posterior cusp. The tips of the paraconid and protoconid of the m1 are broken. A talonid is present on the lingual side of the posterior margin of the m1.

NHMUK PV OR 48929 is a left mandible (figure 4*c,f,i* ). A portion of the mandibular corpus is preserved. Overall, the specimen is pale without marked staining. The anterior edge of a triangular masseteric fossa terminates in line with the midpoint of the m1. The p3 is broken but is evidently double-rooted. The protoconid, paraconid and posterior cusp of the posteriorly inclined p4 are broken but appear to have been well developed. The p4 overlaps with the buccal side of the m1. The paraconid of the m1 is broken but the protoconid is complete. A small talonid is present on the lingual side of the posterior margin of the m1.

NMI NH F18579 is a left mandible (figure 4 *j*–*l*). The corpus is intact and appears robust. The symphysis is rugose and is broken anteriorly (width at break = 20.70 mm) but preserves part of a well-developed mandibular flange, which forms an angle of 135° with the ventral edge of the corpus. A foramen is discernible on the buccal side, anterior of the onset of the flange. The canine alveolus is partially preserved. The coronoid process is broken at the base (anterior–posterior length = 37.32 mm), otherwise, the glenoid to the angle of the dentary is intact. Matrix is adhered to the specimen, which is a brownish-grey colour overall with a mottled appearance that is exacerbated by stria, apparently from chiselling (maximum width of marks is approx. 2.5 mm). These marks are most apparent on the edge of the weathered but triangular masseteric fossa, which terminates at the midline of the m1. The lingual side of the specimen is lighter and less stained than the buccal side. The angle of the dentary, glenoid, remains of the coronoid process and masseteric fossa, are stained orange or red. There is also orange/red staining on the dorsal edge of the corpus, on the symphysis and the remains of the canine alveolus. There is black and dark brown staining on the remainder of the specimen. Remains of p3, p4 and m1 are present. The double-rooted p3 is broken and its dimensions can only be approximated. A small p3–p4 diastema appears to be present. The buccal and lingual faces of the paraconid and protoconid of the posteriorly inclined p4 are only partially preserved. The interface between the posterior edge of the p4 and m1 is a little obscure due to breakage and the presence of matrix, but the limits of the outer margin of the enamel of the p4 are clear ‘in section’. The p4 overlaps the buccal side of the m1. The buccal face of the m1 is broken. The anterior edge is partially preserved; the lingual face is in better condition. A talonid may be present, but it is impossible to say definitively.

### 3.5. Mandibles: comparisons

**Figure 6.**
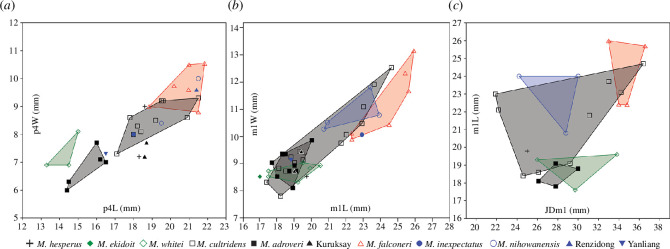
Metric comparisons of the lower dentition in *Megantereon* fossils: (*a*) bivariate plot of raw measurements of fourth lower premolar length (p4L) and width (p4W); (*b*) bivariate plot of raw measurements of first lower molar length (m1L) and width (m1W); and (*c*) bivariate plot of first lower molar length (m1L) and jaw depth (JDm1).

Measurements of the Siwalik mandibles and their dentition indicate that they derived from relatively large forms within the genus (figure 6 *a*–*c*). The mandibular flange in the Siwalik specimens appears well developed and forms an obtuse angle with the ventral edge of the corpus and more similar to group 1 taxa rather than group 2, where this angle is more acute. The ventral edge of the angle of the dentary in NMI NH F18579 is straight and descends at a shallow angle from the corpus, similar to specimens of *M. cultridens*; this edge is curved in *M. nihowanesis*. Conversely, the ventral edge of the angle is relatively short and parallels the long axis of the corpus in the Yanliang *Megantereon*. In specimens of *M. whitei* and *M. adroveri*, this character appears to be more variable.

The p3 of the Siwalik specimens is relatively simple, without developed accessory cusps. In absolute dimensions, the p3 is small in comparison with *M. cultridens* and *M. hesperus* and similar to *M. nihowanensis* and *M. inexpectatus*, but not reduced to the extent observed in *M. adroveri* or *M. whitei*. Small sample size precluded statistical comparisons. In the dataset for the genus, size effects were not detected but were indicated in the group 1 taxa, where third lower premolar length (p3L) was significantly and negatively correlated with size (table 2). Group 2 was not tested due to the small sample size.

The lower fourth premolars (p4) in the Siwalik specimens are comparatively long, high-crowned and robust, with well-developed accessory cusps. Measurements overlap with the upper size range for *M. cultridens,* a specimen of *M. nihowanensis* and the Renzidong *Megantereon* (figure 6*a*). Bivariate statistical testing of the raw values for p4 length and width (p4L and p4W) indicated significant differences between the Siwalik specimens and the group 2 taxa, the group 1 taxa and *M. cultridens*; corrected *p* values for the latter two comparisons, however, were marginal (table 3). Size effects were implicated at the genus level and in group 1; sample size was small in group 2 and examination of size effects was inconclusive (table 2). Bivariate comparison of size-adjusted variables, p4L_adj_ and p4W_adj_, indicated no statistically significant differences between the Siwalik specimens and the group 1 taxa, or the dataset for *M. cultridens* (table 3).

Talonids are commonly, though variably, present on the lower molars in the Siwalik specimens; the diagnostic merits of this character, however, have been questioned [13]. Across the genus, the proportions of the lower carnassial (m1) are much less variable than its upper counterpart. The dimensions of m1 of the Siwalik specimens (m1L and m1W) indicate that while this tooth is relatively long, it is narrow compared with similarly sized congenerics; available data suggests an overlap in absolute dimensions with *M. nihowanensis* and *M. inexpectatus* (figure 6*b*). Bivariate statistical comparisons of the m1 length and width of the Siwalik specimens with group 1, group 2 and *M. cultridens* indicated significant differences (table 3). Strong size effects were implicated in the group 1 taxa; no relationship, however, was indicated in the group 2 dataset (table 2). After adjustment, statistical comparisons of logm1L and m1W_adj_ indicated significant differences between the Siwalik specimens, group 1 taxa and *M. cultridens*.

For multivariate comparisons exceeding two variables, comparative samples were smaller and more taxonomically restricted. The relative size of the Siwalik specimens is illustrated by an examination of jaw depth (JDm1; measured below the anterior edge of the m1 at the alveolus to the ventral edge of the corpus) against the length of the lower carnassial (m1L) (figure 6*c*). Corrected *p*-values for the multivariate comparison of the raw measurements of m1 length, width and jaw depth of the Siwalik specimens indicate significant differences with the genus and group 2 taxa, but not group 1 taxa (*M. cultridens* and *M. nihowanensis*) (table 3). Size effects were implicated at the level of genus, although results were marginal (table 2). Corrected *p*-values for the multivariate comparison of logm1L and size-adjusted values m1W_adj_ and JDm1_adj_ of the Siwalik specimens were only significant for the comparison with the group 2 taxa (table 3).

## 4. Discussion

It is reasonable to infer that the Siwalik fossils are among the largest, if not the largest, known forms of *Megantereon*. Furthermore, within existing paradigms, they display advanced dental characteristics [5,29]. Three notable characters were identified in the upper dentition, which reaffirmed comments by previous workers [22,46]. Firstly, the P3 is reduced in absolute dimensions and in proportion to the P4. No size effects were identified in the proportions of the P3. While it has been suggested that relaxation of selection pressures has resulted in variability in this tooth [18], care must be taken to ‘not assume randomness (or selection) without proof’ [65, p. 424]. A reduction in elements of the dentition with increasing body size is an accepted trend in the evolutionary trajectories of mammalian carnivores [66] and reduction of the P3 in *Megantereon* is regarded as an advanced character [5]. Secondly, a P4 preparastyle is a variable character in *Megantereon* and is better developed and more commonly reported in the closely related, but more derived *Smilodon* [50]. Thirdly, the protocone of the P4 is reduced, resulting in relatively long but narrow dimensions in comparison with congenerics, which is regarded as an advanced character in sabretooth taxa [29]. There appears to be no clear relationship between body size (as inferred from tooth length) and P4 width in *Megantereon*. Given that the proportions of the P4 are regarded as diagnostic, this variability warrants further consideration [18].

The Siwalik mandibles appear large and robust in comparison with their congenerics, but statistical comparison of current samples indicates that they are not significantly so in comparison with larger Eurasian taxa. Morphologically, the mandible appears most similar to *M. cultridens*, although the p3 is simple and somewhat reduced. This may, however, reflect scaling and may be of limited diagnostic significance. The p4 is well developed. Raw measurements of length and width indicate that these dimensions are significantly different from their congenerics. Again, however, body size effects are significant, and the dimensions of this tooth appear to be closely correlated with size. The lower molars in the Siwalik specimens are comparatively narrow relative to their length. Accounting for size effects, non-parametric bivariate comparisons of length and width with the group 1 taxa and with *M. cultridens* indicate that this difference is statistically significant.

Specialization has been shown to constrain morphological variation in hypercarnivores and a logical expectation would be that if variation is observed, it is likely to be minimal, given the functional significance of cheek teeth. It is important to consider potential lineages in *Megantereon* as a continuum rather than a suite of statistically significant differences. Furthermore, the Siwalik fossils should not be regarded as strict contemporaries of one another; they may represent local forms at different points within the Lower Pleistocene. Consistent morphological and metric characters do, however, distinguish the Siwalik specimens as an assemblage from their congenerics and warrant the retention of *M. falconeri* as a distinct morphospecies.

### 4.1. Revised diagnosis

Systematics
Order Carnivora Bowdich, 1821
Family Felidae Batsch, 1788
Subfamily Machairodontinae Gill, 1872
*Megantereon* Croizet and Jobert, 1828
*Megantereon falconeri* (Pomel, 1853)

Lectotype: NHMUK PV OR 16557, partial left mandibular ramus with p4 and m1 *in situ*.

Type locality: Siwalik Hills, India. Pinjor faunal zone, Lower Pleistocene.

Paralectotypes: NHMUK PV OR 39730, fragment of left maxilla with P3–M1 *in situ*; NHMUK PV OR 16554, partial right mandibular ramus with p3–m1 *in situ*; NHMUK PV OR 48929, partial left mandibular ramus with broken p4 and m1 *in situ*; NMI NH F18579, nearly complete left mandible with broken p3, p4 and m1; GSI D-100, partial right mandibular ramus with p3–m1 *in situ*.

Referred specimens: 674 GB 21, partial right mandibular ramus with broken p3, p4 and m1.

A Lower Pleistocene *Megantereon* among the largest of forms in the genus. The third upper premolar and first upper molar are reduced in absolute dimensions and in proportion to the fourth upper premolar. The angle between the lingual edges of the third and fourth upper premolars is obtuse. A preparastyle is present on the upper fourth premolar, which is relatively long but narrow due to the reduction of the protocone. The mandibular flange is well developed and forms an obtuse angle with the ventral edge of the corpus of the mandible, which is robust. The masseteric fossa is triangular, with an anterior margin that terminates at the midline of the first lower molar. The ventral edge of the angle of the dentary is straight and descends at an obtuse angle relative to the corpus. The posterior edge of the angle is concave. The third lower premolar is simple without developed accessory cusps and is single or double-rooted. A small diastema may be present between this tooth and the fourth lower premolar, which is large, high-crowned and posteriorly inclined, with a well-developed posterior cusp. The first lower molar is long but relatively narrow. Talonids are common but variable.

While an exhaustive review of the genus *Megantereon* is beyond the scope of the present paper, the affinities of *M. falconeri* warrant consideration. The conceptual basis of the following model is of morphospecies and is predicated on the assumption of trends, from early to advanced forms, in larger body size and reduction in the relative proportions of anterior premolars (P3 and p3).

It is likely that *M. cultridens* comprised at least two morphotypes: an early small Plio-Pleistocene form (classified in some studies as *M. megantereon* [31,67], followed by a larger form in the Lower Pleistocene. While it is not possible to specify an origin point for the genus with certainty [15], within the confines of this model and available chronological reference, an eastward dispersal of forms is implied. In addition to European specimens, the older morphotype would include the Yanliang mandible and the specimens from Kuruksay. The Renzidong *Megantereon* reflects a divergence from this primitive morphotype and, given similarities in the upper dentition, would represent a Lower Pleistocene form in the lineage that gave rise to *M. inexpectatus*. While it is tempting to also align *M. falconeri* with this lineage, the Siwaliks sabrecat is a Lower Pleistocene form with a much-reduced P3. The Renzidong/*M. inexpectatus* lineage is characterized by an unreduced P3, which is a state that persisted into the Middle Pleistocene.

The second, more advanced morphotype of *M. cultridens* has close affinities with *M. nihowanensis*, which in turn would represent an advanced form in this lineage and is distinguished by increased size and reduction in the absolute and proportional dimensions of the P3. While also of large size, the proportions of the carnassial teeth and reduction of the P3 distinguish *M. falconeri* from this advanced *M. cultridens* and *M. nihowanensis*. A parsimonious scenario, then, would be that *M. falconeri* developed in parallel to, or was an early divergence from (as is hypothesized for *M. adroveri*), the advanced morphotype of *M. cultridens*.

Whether this model and these criteria have any phylogenetic merit is of course open to question. It is reasonable to suggest that differences in the Siwalik specimens simply reflected localized, functional adaptations. *Megantereon falconeri* is arguably the largest known form within the genus. Given that large size facilitates the capture of large prey and that the carnassial morphology of this morphospecies probably reflected selection for the rapid access to and the efficient removal of soft tissue, it is reasonable to hypothesize a comparative (in the context of the genus) specialization on larger bodied prey. The Pinjor faunal zone of the Upper Siwaliks is host to a suite of potential prey animals [35]. While allowing for the constraints of sample size and broad temporal resolution, revisiting and comparing the palaeoecological contexts of *Megantereon* spp., including the relatively poorly understood carnivore guild of the Siwaliks, may also be a fruitful line of enquiry to consider the significance of morphological characters and for furthering our understanding of these extinct predators.

## 5. Conclusion

The differentiation of fossils of closely related carnivorous mammals can be challenging. Morphological diversity in craniodental characters may be restricted by functional constraints and potentially confounded by allometric effects. The question of the number and affinities of morphospecies of the extinct sabretooth cat genus *Megantereon* is the subject of long-standing debate and the taxonomic status and affinities of fossils from the Siwaliks in South Asia have been unclear.

Here, review and qualitative and quantitative comparisons with congenerics, including investigation and controls for allometric effects, indicate that the Siwalik *Megantereon* fossils represent a distinct morphospecies and the status of *M. falconeri* is upheld. *Megantereon falconeri* is among the largest, if not the largest, of forms in the genus and within existing paradigms, displays advanced dental characteristics. Through comparisons of phenotypic characters, it is hypothesized that while there are similarities between East Asian specimens it is proposed that *M. falconeri* derived from, or developed in parallel to, an advanced Lower Pleistocene form of the western Eurasian species *M. cultridens*.

## Data Availability

Data provided in article or uploaded as electronic supplementary material [68].
